# Surface and Antimicrobial Properties of Ester-Based Gemini Surfactants

**DOI:** 10.3390/molecules30122648

**Published:** 2025-06-19

**Authors:** Iwona Kowalczyk, Adrianna Szulc, Anna Koziróg, Anna Komasa, Bogumił Brycki

**Affiliations:** 1 Department of Bioactive Products, Faculty of Chemistry, Adam Mickiewicz University Poznan, 61-614 Poznan, Poland; iwkow@amu.edu.pl (I.K.); adrszu@gmail.com (A.S.); aniak@amu.edu.pl (A.K.); 2 Institute of Fermentation Technology and Microbiology, Faculty of Biotechnology and Food Sciences, Lodz University of Technology, 90-530 Lodz, Poland; anna.kozirog@p.lodz.pl

**Keywords:** ester-based gemini surfactants, betainate gemini surfactants, antimicrobial activity, DFT calculations

## Abstract

Cationic surfactants, accounting for approximately 7% of the global surfactant market, are widely used in applications such as fabric softeners, biocides, and corrosion inhibitors. Recently, gemini surfactants—comprising two amphiphilic units linked by a spacer—have attracted significant interest due to their superior surface activity, lower critical micelle concentrations, and strong antimicrobial properties. However, their poor biodegradability, resulting from their complex molecular structure, has raised environmental concerns. To address this, researchers have developed ester-based gemini surfactants incorporating biodegradable bonds. This study aimed to investigate the relationship between the structure of ester-based gemini surfactants (hydrophobic chain length and spacer type) and their antimicrobial activity against bacteria and fungi. Three series of compounds featuring different functional groups in the spacer were synthesized, along with a trimeric surfactant for comparative purposes. The results demonstrated that both the hydrophobic chain length and the presence of additional cationic groups significantly influence the CMC and antimicrobial performance. Quantum mechanical calculations were also performed to search for correlations between electronic properties and chemical reactivity of compounds. These findings highlight that ester-based gemini surfactants combine high surface and antimicrobial activity with the potential for improved biodegradability, making them promising candidates for use in environmentally friendly applications.

## 1. Introduction

Cationic surfactants, which constitute nearly 7% of the global surfactant market, are utilized in a wide range of applications, including fabric softeners, asphalt modifiers, corrosion inhibitors, biocides, and textile processing agents [1]. In recent years, gemini surfactants—a new class of cationic surface-active agents—have garnered significant interest. These compounds are generally composed of two amphiphilic units linked by a spacer molecule [2,3,4,5]. There is a growing body of research focused on their synthesis and the exploration of their remarkable and often unconventional properties. Compared to traditional monomeric surfactants, gemini surfactants are much more efficient at reducing interfacial tension and can form micelles at notably low critical micelle concentrations (CMC) [6,7,8,9]. Additionally, they offer enhanced wetting characteristics and exhibit unique rheological and aggregation behavior. Owing to these advantageous features, gemini surfactants are being investigated for a wide range of potential applications, including enhanced oil recovery [10,11,12], gene delivery systems [13,14,15], corrosion prevention in iron-based materials [16,17,18], and environmental remediation [19,20].

Moreover, gemini surfactants exhibit strong antimicrobial activity due to their unique molecular structure, which enhances their ability to disrupt microbial cell membranes. The presence of two cationic head groups and hydrophobic tails enables more effective interaction with negatively charged bacterial surfaces, leading to increased membrane permeability and cell lysis. These compounds have shown broad-spectrum activity against both Gram-positive and Gram-negative bacteria, as well as fungi [16,21,22,23,24]. Their high surface activity and low CMC contribute to their efficiency at low dosages, making them promising candidates for use in disinfectants, personal care products, and biomedical applications [15].

**Figure 1:**
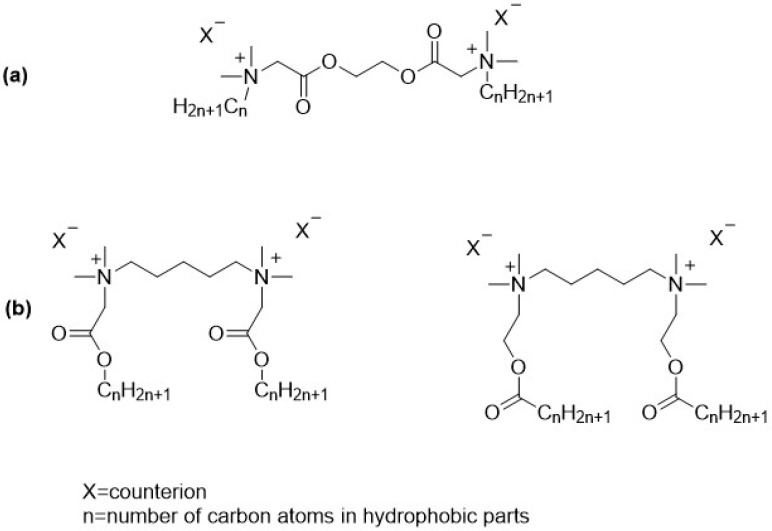
Example structures of ester-based gemini surfactants: (**a**) ester bonds in spacer; (**b**) ester bonds in hydrophobic parts of the molecules (first one—betainate, second—esterquat gemini).

Gemini surfactants are generally considered to be poorly biodegradable, largely due to their complex molecular structure and the presence of multiple hydrophobic and cationic groups. This resistance to microbial degradation poses environmental concerns, especially for applications involving large-scale or long-term use [25,26]. To address this issue, researchers are developing modified gemini surfactants incorporating biodegradable linkers or more environmentally friendly components to enhance their breakdown in natural environments. Nonetheless, environmental considerations are becoming increasingly important in the development of new surfactant systems. With stricter environmental policies and growing ecological awareness, the demand for eco-friendly alternatives to conventional surfactants is on the rise. One promising approach to improving biodegradability is the incorporation of labile bonds, such as ester linkages, into surfactant molecules [27,28,29]. Ester-based gemini surfactants can be designed with various structural configurations to tailor their properties and biodegradability. Ester bonds may be introduced into the hydrophobic tails [26,30], the spacer linking the two head groups, or as part of side chains [31,32,33] (Figure 1). Compounds containing an ester group in both the linker and the substituents are also described in the literature [34], but they are much more difficult to obtain and, for now, have no major practical significance. Each arrangement influences the surfactant’s stability, hydrolysis rate, and surface activity.

The aim of the present work was to investigate the relationship between the structure of the ester-based gemini surfactants (hydrophobic chain length) and antimicrobial activity against bacteria and fungi. Another criterion differentiating the classes of tested gemini surfactants is the structure of the linker. In this article, we present the antimicrobial activity of compounds containing a functionalized spacer with an amine or ether group. Additionally, for comparative purposes, a compound containing three ammonium groups was obtained and analyzed.

## 2. Results

### 2.1. Synthesis

**Figure 2:**
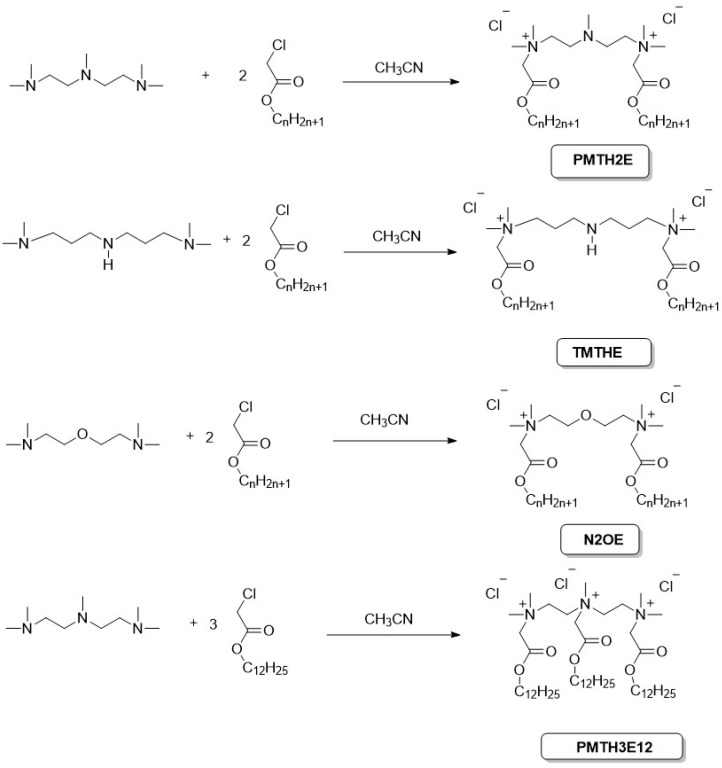
Synthesis of obtained surfactants.

We obtained three series of ester-based (betainate type) gemini surfactants: PMTH2 compounds possess an additional tertiary amino group in the linker, TMTH compounds have additional secondary amino group in the linker and N2OE compounds possess additional ether group in the spacer. Additionally, for comparative purposes, a trimeric compound was obtained, designated as PMTH3. All gemini surfactants were obtained in our laboratory by quaternization of the appropriate tertiary diamines with the appropriate acyl chlorides (Figure 2).

**Figure 3:**
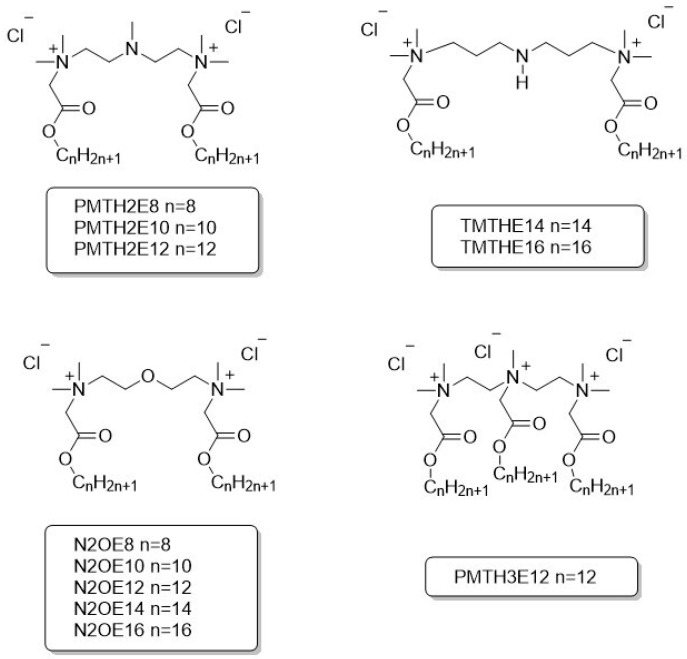
Structures and abbreviations of ester-based gemini and trimeric surfactants.

All compounds were obtained with a purity above 90%. The synthetic details and spectroscopic data were described earlier: PMTH2 and TMTH [27,35], N2OE [36]. The formulas and abbreviations of compounds analyzed in this article are presented in Figure 3.

### 2.2. Surface Properties of Gemini Surfactants

Gemini surfactants exhibit significantly higher surface activity than their monomeric counterparts. Due to their dual hydrophilic head groups and hydrophobic tails connected by a spacer, they adsorb more efficiently at interfaces, reduce surface and interfacial tension more effectively, and form micelles at much lower CMCs [2,37,38]. Ester-based gemini surfactants are an attractive alternative to classic gemini surfactants because they have lower CMC values [39]. In the case of gemini ester derivatives, one can also observe their significant advantage in surface properties in relation to monomeric surfactants with the same length of hydrophobic substituent [40,41].

**Figure 4:**
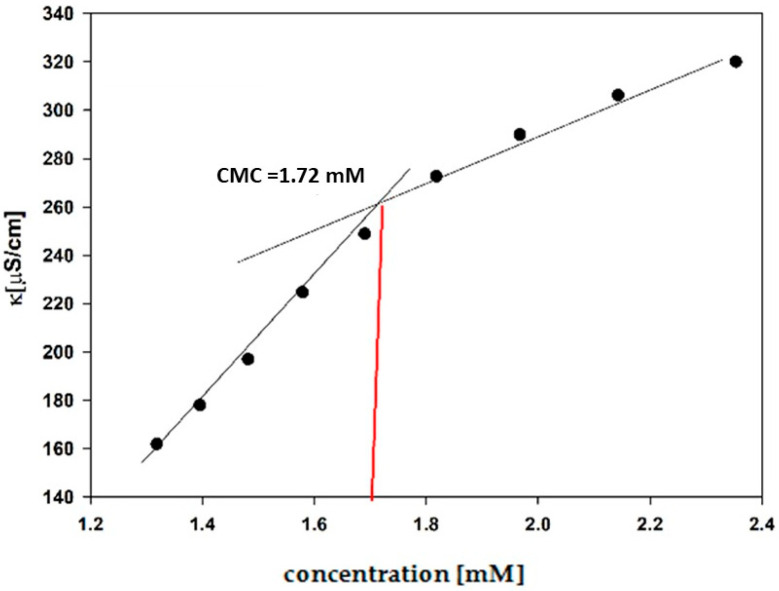
A plot of specific conductivity (k) for the aqueous solution of N2OE10 versus its concentration.

The CMC values for all synthesized surfactants were obtained by conductometric titration. The exemplary relationship of the specific conductivity (k) on the concentration is shown in Figure 4.

The values of CMC, degree of counterion binding (β), and standard Gibbs energy of micellization (ΔG°mic) for all of obtained surfactants are listed in Table 1. The CMC values of ester-based gemini surfactants, similar to other gemini surfactants, decrease with the increase in the number of methylene groups in the hydrocarbon chain and the increase in the number of positively charged nitrogen atoms [42].

The aggregation parameters in Table 1 illustrate clear trends in the self-assembly behavior of ester-based gemini and trimeric surfactants. For the PMTH2En series, a gradual decrease in the CMC is observed with increasing hydrophobic chain length: from 2.1 mM (PMTH2E8) to 1.6 mM (PMTH2E12). This indicates enhanced micelle formation as the hydrophobic chain becomes longer. The counterion binding parameter (β) increases from 0.20 to 0.53, suggesting that longer chains promote stronger counterion association. The ΔG°mic becomes more negative, shifting from −23.09 to −34.56 kJ/mol, confirming that micellization is thermodynamically more favorable for surfactants with longer hydrophobic chains. The trimeric surfactant PMTH3E12 shows a remarkable reduction in CMC (0.013 mM), highlighting the significant impact of an additional cationic head group. Its ΔG°mic is also more negative (−51.86 kJ/mol), indicating highly spontaneous micelle formation. The TMTHE series follows the expected pattern: CMC decreases from 0.08 mM (TMTHE14) to 0.03 mM (TMTHE16), while ΔG°mic becomes more negative, demonstrating stronger micellization with longer chains. For the N2OEn series, the initial CMC is relatively high at 8.50 mM (N2OE8), but decreases significantly to 0.05 mM (N2OE16). The β values are higher across this series (up to 0.63), suggesting strong counterion binding. The ΔG°mic values also become more negative as the chain length increases from −34.77 to −57.14 kJ/mol. The results confirm that both increasing hydrophobic chain length and introducing additional cationic head groups improve micellization efficiency, reduce CMC, and enhance thermodynamic favorability of micelle formation.

### 2.3. Antimirobial Activity

Gemini surfactants with ester bonds are also known from their antimicrobial activity [43,44,45]. All obtained surfactants were subjected to tests to determine antimicrobial activity. The obtained values of the minimum inhibitory concentration (MIC) are listed in Table 2.

Taking into account bacteria and microscopic fungi as a common pool of microorganisms, it can be stated that the most effective antimicrobial compounds in the group tested are PMTH2E8 and PMTH2E10. For all tested microorganisms, the MIC value is equal to or lower than 156 mM. The highest MIC values were obtained for compounds containing 14 and 16 methylene groups (TMTHE14, TMTHE16, N2OE14, N2OE16). Gemini surfactants with shorter hydrophobic chains, where the number of -CH_2_- groups is between 8 and 12, show better biocidal efficacy due to the optimal combination of hydrophobicity, solubility, and flexibility. These features allow for effective adsorption and destabilization of microbial membranes, which translates into lower concentrations inhibiting the growth of microorganisms. The authors have already proven the relationship between the MIC value and the length of the alkyl chain in previous studies [37,46], which was also shown by [47,48,49]. The length of the alkyl chain is only one of the elements influencing antimicrobial efficacy. An additional element, presented for the first time in this study, is the insertion of ester groups into the chain structure, which strongly influence value of minimal concentration, inhibiting growth of microorganisms.

**Figure 5:**
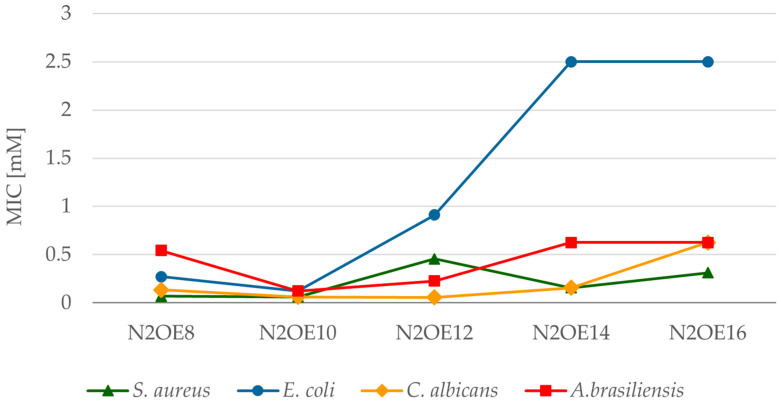
The relationships between MIC and the number of carbon atoms in the alkyl substituent for N2OEn (*n* = 8, 10, 12, 14, 16).

However, it should be remembered that bacteria and microscopic fungi are microorganisms with two completely different cell structures. Without a doubt, such a difference between the prokaryotic cell in bacteria and the eukaryotic cell in fungi influences the different effects of biocides. For this reason, the values of the MIC of individual groups of microorganisms are also different. All compounds containing twelve-carbon chains are characterized by lower MIC values for microscopic fungi compared to bacteria. The sensitivity range of the tested microorganisms for the compounds PMTH2E12, PMTH3E12, and N2OE12 is as follows: *Candida albicans* < *Aspergillus brasiliensis* < *Staphylococcus aureus* < *Escherichia coli*. The least sensitive to the action of these compounds are therefore Gram-negative bacteria *E. coli*. In turn, compounds containing 8 and 10 carbon atoms in the aliphatic chains PMTH2E8, PMTH2E10, N2OE8, and N2OE10, as well as 14 and 16 carbon atoms—TMTHE14, TMTHE16—at the lowest concentrations acted on Gram-positive bacteria *Staphylococcus aureus* and yeast *Candida albicans*. The least sensitive to these compounds were the molds, Aspergillus brasiliensis. The sensitivity series is slightly different for compounds N2OE14 and N2OE16 (Figure 5), which contain oxygen in the spacer instead of a nitrogen atom. In this case, the most sensitive are Gram-positive bacteria, followed by microscopic fungi, and the least sensitive are Gram-negative bacteria.

In the obtained results, the compound PMTH3E12 deserves attention, as it contains three hydrophobic chains in the molecule. This compound acts at a concentration over 16 times lower on *A. brasiliensis* molds compared to *E. coli* bacteria. This is influenced, among other factors, by the structure of external structures—the cell wall and membrane, which are the first place of contact of biocidal compounds with the cell. Gram-positive bacteria such as *S. aureus* have a thick layer of peptidoglycan in their cell wall, and Gram-negative bacteria such as *E. coli*—additionally an external lipid membrane. These structures may hinder the penetration of large, strongly hydrophobic molecules, such as three-chain surfactants. Fungi, on the other hand, contain membranes in their cellular structure, which contain more lipids, including sterols with the main compound—ergosterol. Due to such a cellular structure, microscopic fungi are more susceptible to the effects of hydrophobic surfactants. Additionally, the antifungal activity of the PMTH3E12 compound is better compared to the PMTH2E12 analog, containing two hydrophobic chains. Three-chain surfactants have a larger hydrophobic surface, which allows for a stronger effect and better penetration of the lipid cell membrane of fungi. This leads to much greater destabilization of the membrane, disruption of its integrity, lysis or ion efflux [49,50,51,52].

In the presented work, ester bonds -COO- were introduced into the structure of the hydrophilic chain, which are connected to the nitrogen atom by a methylene group -CH_2_-. Such gemini surfactants with betaine groups have a changed structure of the polar head, which affects their ability to aggregate and interact with cell membranes. These compounds are more hydrophilic, compared to classic gemini alkyl surfactants, which reduces their ability to interact with lipid cell membranes of microorganisms. As a result, betaine surfactants may show a weaker ability to destabilize bacterial and fungal membranes, which translates into a decrease in antimicrobial activity. However, comparing the MIC values obtained in the presented work with the values obtained in earlier studies [37,46], where compounds containing simple alkyl chains were used, the described trend is variable. Compounds containing only 8 to 10 carbon atoms in the alkyl chain show lower MIC values of ester-based gemini surfactants. This tendency changes when the chain length increases to 14–16 methylene groups. The MIC value of compounds containing the simple alkyl chain 14-O-14 increases for *E. coli* by up to more than 80-fold compared to N2OE14 (from a value of 0.0293 mM to 2.5 mM, respectively). In turn, for compounds containing 12 methylene groups in the chain, the MIC value for the tested microorganisms is variable and strictly depends on the compound—its linker structure and counterions. In the presented work, classical gemini surfactants containing only alkyl chains were replaced with ester derivatives because they are more easily biodegradable [27], which is extremely important from the point of view of environmental protection and the quantities in which biocides are currently used.

### 2.4. Quantum Mechanical Calculations

**Figure 6:**
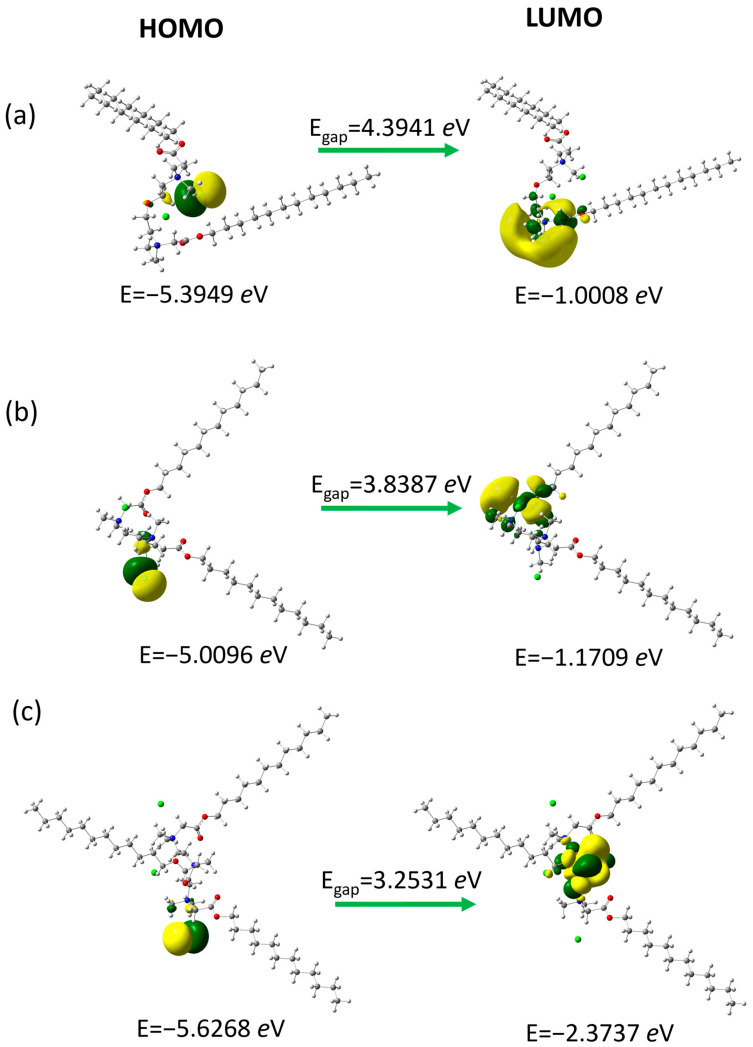
Frontier molecular orbitals for (**a**) N2OE12, (**b**) PMTH2E12, and (**c**) PMTH3E12. Green and yellow colors represent opposite signs of the orbital values.

We have analyzed the frontier molecular orbitals (Figure 6), as they play an important role in the chemical reactivity of molecules. Representatives of surfactant groups with a twelve-carbon chain and different linker structures were selected for analysis using quantum mechanical calculations to estimate the dependencies resulting from the linker structure: with an ether group (N2OE12), an additional tertiary (PMTH2E12), and an additional quaternary amine group in the linker (PMTH3E12). These orbitals participate in chemical reactions or interactions with other species and indicate the active site of the compound.

The highest occupied molecular orbital (HOMO) with higher energy is an electron-rich molecular orbital. It acts primarily as an electron donor and is responsible for the nucleophilicity of the molecule. In studied compounds, it is located around the chloride anion. The lowest unoccupied molecular orbital (LUMO) with lower energy is characterized by electron deficiency and the ability to accept electrons, which is related to the electrophilicity of the molecule. It is distributed over the fragments of the linker near the nitrogen atoms. The so-called energy gap (E_gap_), which is the difference between E_LUMO_-E_HOMO_ is related to the chemical reactivity and kinetic stability of the molecules [53]. Moreover, it explains the charge transfer interaction within the molecular moieties, which influences the biological activity of compounds [54,55]. The calculated energy gap increases in the order PMTH3E12 < PMTH2E12 < N2OE12 (Table 3).

Additionally, based on the values of the HOMO and LUMO energies, the global reactivity parameters related to the chemical reactivity and stability of the compounds were calculated, i.e., the chemical potential (μ = (E_LUMO_ + E_HOMO_)/2), global hardness (η = (E_LUMO_ − E_HOMO_), global softness (σ = 1/2η), global electrophilicity index (ω = μ2/2η) and the maximum electronic charge (ΔN_max_ = −μ/η) [56,57,58]. Electronic chemical potential μ and global hardness η tend to increase while global softness σ, global electrophilicity ω, and global maximum electron transfer ΔN_max_ tend to decrease in the order, PMTH3E12, PMTH2E12, and N2OE12. Hardness and softness are related to the reactivity and stability of compounds. The lower value of hardness in a molecule refers to a soft molecule and is related to higher chemical reactivity. The lowest hardness value is shown by PMTH3E12, which also has the best antifungal properties. As already mentioned, bacteria and microscopic fungi have different cell structures, so inverse correlations between global reactivity parameters and antibacterial and antifungal properties can be expected.

**Figure 7:**
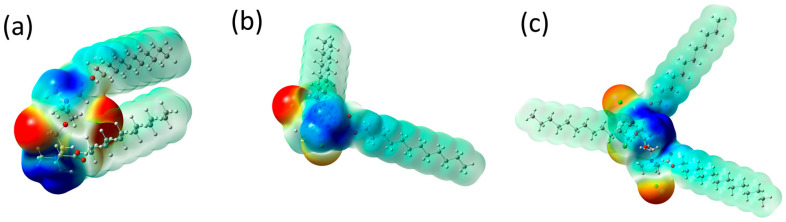
Molecular electrostatic potential maps for (**a**) N2OE12, (**b**) PMTH2E12, and (**c**) PMTH3E12. Red and blue colors denote to the negative and positive electrostatic potential, respectively. Light green color indicates the neutral electrostatic potential.

Based on the electrostatic potential maps, trends in electrostatic interactions of surfactant molecules can be indicated (Figure 7). The negative electrostatic potential, marked in red, corresponds to concentrated electron density (Cl^−^ counterions) in the molecules. In contrast, the positive electrostatic potential, the bluest area, indicates strongly positive fragments of the molecules (quaternary nitrogen atoms). Light green color indicates the neutral electrostatic potential of hydrocarbon chains, which is associated with the dominant role of the van der Waals effect in interactions, including dispersion and exchange-repulsion potentials [59].

## 3. Materials and Methods

Gemini and trimeric surfactants were obtained according to the procedure development in our laboratory [27,35,36]. All reagents and solvents were obtained from Merck (Poznan, Poland).

CMC values were obtained conductometrically by using a Conductivity Meter Elmetron CC-505 (Zabrze, Poland). The apparatus was calibrated by using a standard (147 μS/cm in 298.15 K). All the solutions were prepared using double-distilled water. Conductivity measurements were carried out at a temperature of 298.15 K. The conductometric titration was repeated at least three times for each gemini surfactant, and CMC was calculated as the mean value of three measurements. The CMCs of synthesized compounds were obtained by conductometric titration, creating relationship graphs of the characteristic conductivity in water of the surfactants as a function of the concentration [37]. The graphs consist of two lines with differing slopes. The line with higher inclination shows behavior before micellization, and the second line illustrates the process of micellization. The CMC values are at the intersection of the linear regressions of these lines. The degree of counterion binding (β) was calculated according to Frahm’s method [60] as (1 − α), where α = Smicellar/Spremicellar, i.e., the ratio of the slope after and before CMC. The ΔG°mic values were calculated by using Equation (1) [37]:

(1) $$\Delta G_{CMC}^{0}=2RT\left(\frac{1}{2}+\beta \right)lnCMC-RTln2$$

where R is the gas constant, T is the temperature in K, and the CMC is in mol/L.

The ester-based gemini and trimeric surfactants were tested for antimicrobial activity against bacteria: *Escherichia coli* ATCC 10536, *Staphylococcus aureus* ATCC 6538, yeast *Candida albicans* ATCC10231, and molds *Aspergillus niger* ATCC 16401. The MIC values for all microorganisms were determined by a tube standard two-fold dilution method [21]. Each of microorganisms was resuspended in physiological salt solution (molds in water with Tween 80 addition) and diluted to 10^7^ cfu/mL for bacteria and 10^6^ cfu/mL for yeast and molds. In the next step, 1 mL of microorganism suspension was mixed with 1 mL of media: TSB (Merck Poznan, Poland) for bacteria/MEB (Merck Poznan, Poland) for microscopic fungi containing serial dilutions of the tested compounds. All samples were incubated at 37 °C for 24 h bacteria, 48 h yeast, and 28 °C for 48 h molds. As a growth control, a suspension of microorganisms in a medium without the biocides was used. The MICs were defined as the lowest concentration of the compounds in which there was no visible growth.

The DFT calculations were performed using the GAUSSIAN16 program package [61]. The calculations employed the B3LYP exchange–correlation functional, which combines the hybrid exchange functional of Becke [62,63] with the gradient-correlation functional of Lee et al. [64] in conjunction with the 6–311 ++ G(d,p) basis set [55]. The HOMO and LUMO energies were calculated and the energy value from a.u. was converted to eV (1 a.u. = 27.2114 eV).

## 4. Conclusions

In this study, a series of ester-based gemini and trimeric surfactants with varying hydrophobic chain lengths and spacer structures were synthesized and characterized. The aggregation behavior revealed that increasing the alkyl chain length and the number of cationic head groups significantly enhances micellization efficiency, as evidenced by lower CMC, higher counterion binding parameters, and more negative ΔG°mic. The antimicrobial activity tests demonstrated that these surfactants possess broad-spectrum efficacy against Gram-positive and Gram-negative bacteria as well as fungi, with notable differences depending on both chain length and molecular architecture. Interestingly, shorter-chain derivatives generally exhibited stronger antimicrobial effects at lower concentrations, whereas trimeric and long-chain surfactants showed enhanced surface activity but slightly reduced antimicrobial potency. Quantum mechanical calculations provided insights into the electronic structure and reactivity of selected compounds, highlighting how molecular features influence their physicochemical and biological properties. The data suggest that compounds with smaller energy gaps and lower global hardness exhibit higher chemical reactivity, which may correlate with their biological activity. Overall, this work underscores the potential of ester-based gemini and trimeric surfactants as multifunctional agents with tunable properties, suitable for various industrial and biomedical applications. Future research should focus on further improving their biodegradability to align with environmental sustainability goals.

## Figures and Tables

**Table 1:** Aggregation parameters for ester-based gemini and trimeric surfactants.

Compounds	CMC [mM]	β	ΔG°mic [kJ/mol]
PMTH2E8	2.1 ± 0.1	0.20	−23.09
PMTH2E10	1.8 ± 0.1	0.40	−29.89
PMTH2E12	1.6 ± 0.1	0.53	−34.56
PMTH3E12	0.013 ± 0.004	0.40	−51.86
TMTHE14	0.08 ± 0.02	0.48	−47.50
TMTHE16	0.03 ± 0.01	0.52	−54.33
N2OE8	8.50 ± 0.11	0.90	−34.77
N2OE10	1.72 ± 0.09	0.71	−39.86
N2OE12	0.12 ± 0.04	0.57	−49.56
N2OE14	0.09 ± 0.01	0.61	−52.93
N2OE16	0.05 ± 0.01	0.63	−57.14

**Table 2:** MIC values of ester-bonded gemini and trimeric surfactants.

Compounds	MIC [mM]			
	*S. aureus* ATCC 6538	*E. coli* ATCC 10536	*C. albicans* ATCC 10231	*A. brasiliensis* ATCC 16404
PMTH2E8	0.0195	0.039	0.0195	0.156
PMTH2E10	0.0195	0.039	0.0195	0.156
PMTH2E12	0.447	0.895	0.056	0.224
PMTH3E12	0.651	1.302	0.0407	0.081
TMTHE14	0.625	2.5	0.625	2.5
TMTHE16	1.25	2.5	0.625	2.5
N2OE8	0.068	0.272	0.136	0.544
N2OE10	0.062	0.124	0.062	0.124
N2OE12	0.456	0.911	0.057	0.228
N2OE14	0.156	2.5	0.156	0.625
N2OE16	0.3125	2.5	0.625	0.625

**Table 3:** Global reactivity parameters (in eV) of the N2OE12, PMTH2E12, and PMTH3E12.

	N2OE12	PMTH2E12	PMTH3E12
E_HOMO_	−5.3949	−5.0096	−5.6268
E_LUMO_	−1.0008	−1.1709	−2.3737
E_gap_	4.3941	3.8387	3.2531
µ	−3.1979	−3.0903	−4.0002
η	2.1971	1.9194	1.6266
σ	0.2276	0.2605	0.3740
ω	2.3273	2.4877	4.9189
ΔN_max_	1.4555	1.6100	2.4592

µ = electronic chemical potential; η = global hardness; σ = global softness; ω = global electrophilicity index; ΔN_max_ = maximum charge transfer index.

## Data Availability

The data presented in this study are available in the article.

## Abbreviations

The following abbreviations are used in this manuscript:

CMC	critical micelle concentration
MIC	minimum inhibitory concentration
FMOs	frontier molecular orbitals
HOMO	highest occupied molecular orbital
LUMO	lowest unoccupied molecular orbital
